# Integrated Approach to Highlighting the Molecular Bases of a Deep Vein Thrombosis Event in an Elite Basketball Athlete

**DOI:** 10.3390/ijms241512256

**Published:** 2023-07-31

**Authors:** Cristina Mennitti, Ciro Miele, Carmela Scarano, Iolanda Veneruso, Alessandro Gentile, Rosaria Mormile, Francesca Saviano, Giovanni D’Alicandro, Cristina Mazzaccara, Giulia Frisso, Filomena Capasso, Valeria D’Argenio, Olga Scudiero

**Affiliations:** 1 Department of Molecular Medicine and Medical Biotechnology, University of Naples Federico II, 80131 Naples, Italy; cristinamennitti@libero.it (C.M.); venerusoi@ceinge.unina.it (I.V.); olga.scudiero@unina.it (O.S.); 2 UOC Laboratory Medicine, Hematology and Laboratory Haemostasis and Special Investigations, AOU Federico II University of Naples, 80131 Naples, Italy; 3 CEINGE-Biotecnologie Avanzate Franco Salvatore, Via G. Salvatore 486, 80145 Naples, Italy; 4 Hematology, Department of Translation and Precision Medicine, Sapienza University, Via Benevento 6, 00161 Rome, Italy; 5 Department of Neuroscience and Rehabilitation, Center of Sports Medicine and Disability, AORN, Santobono-Pausillipon, 80122 Naples, Italy; 6 Department of Human Sciences and Quality of Life Promotion, San Raffaele Open University, Via di Val Cannuta 247, 00166 Rome, Italy; 7 Task Force on Microbiome Studies, University of Naples Federico II, 80100 Naples, Italy

**Keywords:** laboratory medicine, molecular analyses, physical exercise, thrombophilia, von Willebrand factor, coagulation, calreticulin

## Abstract

Acute or intense exercise can result in metabolic imbalances, muscle injuries, or reveal hidden disorders. Laboratory medicine in sports is playing an increasingly crucial role in monitoring athletes’ health conditions. In this study, we designed an integrated approach to explore the causes of a deep venous thrombosis event in an elite basketball player. Since the complete blood count revealed a marked platelet count (838 × 10^3^ µL), and thrombophilia screening tests did not reveal any significant alteration, we evaluated the thrombin generation, which highlights a state of hypercoagulability. First-level haemostasis exams showed only a slight prolongation of the activated Partial Thromboplastin Time (aPTT). Thus, screening tests for von Willebrand Disease showed a reduction in vWF parameters. Therefore, we directed our hypothesis towards a diagnosis of acquired von Willebrand disease secondary to Essential Thrombocythemia (ET). To confirm this hypothesis and highlight the molecular mechanism underlying the observed phenotype, molecular tests were performed to evaluate the presence of the most common mutations associated with ET, revealing a 52-bp deletion in the coding region of *CALR* exon 9. This case report highlights the importance of an integrated approach to monitoring the athletes’ health status to personalise training and treatments, thus avoiding the appearance of diseases and injuries that, if underestimated, can undermine the athlete’s life.

## 1. Introduction

Although physical exercise and an appropriate diet represent a correct lifestyle capable of preventing the appearance of some pathologies, such as heart disease [1], stroke [2], diabetes [3], and breast and colon cancer [4], the practice of physical activity can have a double effect. Indeed, mild to moderate aerobic exercise can be beneficial to health and can also increase life expectancy [5]. On the other hand, intense effort could promote the occurrence of specific conditions, such as hereditary or congenital cardiovascular diseases [6], thrombotic events [7], or metabolic defects [8], which may not be evident, especially in the absence of family history and symptoms. For this reason, the monitoring and identification of specific biochemical and/or molecular parameters can represent a valid support tool in the evaluation of the general state of health of the athlete [9].

In particular, although exercise is thought to have a protective role against thrombosis as a result of the controlled balance between exercise-activated coagulation and fibrinolytic pathways [10], recent studies have demonstrated a negative correlation between sport activity and thrombotic events. Indeed, deep venous thrombosis (DVT) is the result of three different mechanisms: venous stasis, hypercoagulability, and endothelial dysfunction, also known as Virchow’s triad [11]. Each of these factors, alone or combined, may appear in an athlete depending on the intensity and duration of exercise. In addition, athletes are exposed to many acquired risk factors, including trauma, immobilization, and long-term travel, that may predispose them to thrombotic events. Thrombosis of the deep veins of the upper limb after strenuous exercise or trauma is a rare but well-recognised condition, often called Paget–Schroetter’s syndrome or effort thrombosis, and in most cases, it affects the axillary and subclavian veins. A potentially lethal complication is pulmonary embolism, which may occur in 36% of patients. On the other hand, lower limb venous thrombosis appears post-trauma mostly at the level of the popliteal vein, posterior tibial vein, and peroneal vein. Clinically, the patient presents unilateral oedema of the lower limb, particularly visible on the calf and ankle; tension or pain in the calf; moderate fever; and positive Homans sign (calf pain on leg dorsiflexion) [12]. The potential complications are pulmonary embolism and post-thrombotic syndrome (PTS). Pulmonary embolism may occur in 50% of untreated DVT patients and may cause death in 2.1% of cases.

It has to be underlined that inherited diseases may also alter the coagulation status of an individual and be responsible for a deep venous thrombosis event. In this context, essential thrombocythemia (ET) is a rare Philadelphia-negative chronic disease characterised by an uncontrolled proliferation of atypical megakaryocytes in the bone marrow. It belongs to a group of haematological disorders called Myeloproliferative Neoplasms (MPN), which include, in addition to ET, also Polycythemia Vera (PV) and Primary Myelofibrosis. The common denominator of MPN is the presence of mutations (known as driver mutations) constitutively activating the JAK-STAT signalling transduction pathways in the haematopoiesis [13]. Mutated genes responsible for ET are Janus kinase 2 (JACK2 V617F), Calreticulin (CALR) and Thrombopoietin Receptor (TPO-R) *MPL* (with a frequency of 60%, 20%, and 3%, respectively). There is a percentage of patients who do not present any of the three driver mutations (about 10–20%), hence are called Triple Negative [14]. Assessment of patients affected by ET comprises a complete blood count, a bone marrow biopsy, and genetic analysis to assess the existence of genetic alterations causing ET. Due to the similarity of symptoms in myeloproliferative neoplasms, it is crucial to exclude alternate reasons for thrombocytosis, such as clonal and reactive factors, prior to establishing a conclusive diagnosis of essential thrombocytosis [15]. Acquired von Willebrand syndrome (AVWS) is a rare bleeding disorder most commonly observed in patients with essential thrombocythemia (ET). The main factors distinguishing AVWS from the congenital form of von Willebrand disease encompass the absence of previous bleeding disorders, a diagnosis later in life, and a negative family history [16,17]. AvWS often occurs secondary to underlying disorders like cardiovascular disorders, lymphoproliferative disorders, hematologic and solid malignancies, and autoimmune disorders [18].

Considering all the above, in recent years, laboratory medicine applied to sports has assumed an essential role in monitoring athletes’ health status. In fact, it provides valuable information to coaches and sports doctors who establish personalised training and recovery programs and, if necessary, provide nutritional supplements to athletes in order to improve their athletic performance and reduce the decline in performance and/or risk of muscle injury [19,20]. In this scenario, we described a case report of a young basketball athlete in which an integrated approach made of biochemical and haematological dosages, as well as genetic investigations, allowed us to identify the molecular mechanisms responsible for a single thrombotic event reported by the same athlete.

## 2. Results

### 2.1. Clinical History

Here, we report the case of a 28-year-old competitive athlete who came to our attention because he was part of the basketball team monitored by our team for 5 years [19]. The athlete was admitted to our Haematology Department following a thrombotic event, and he did not report the use of any anticoagulant drugs or other medications at the time of the event. The athlete referred to an “A” blood type and stated that he suffered an occlusive left femoral vein thrombus with no impairment to perfusion or motor function present on evaluation. He denied any family history of cardiovascular diseases or sudden cardiac death in the first-degree relatives. He was not a smoker and denied using drugs or other substances. As supported by the current guidelines [21,22], the athlete underwent a standard clinical evaluation consisting of family and personal history, pre-participation examination, electrocardiogram (ECG), and stress tests. The medical examination revealed that the athlete was rated suitable for competitive sporting practice.

### 2.2. Clinical Laboratory Determinations

To evaluate the general health status of the athlete, we performed biochemical and haematological determinations, and the results show that all are within reference values, except for red blood cell and platelet counts. In Table 1, we have reported only a few of the analysed parameters, i.e., those most suggestive of the athlete’s health conditions.

### 2.3. Coagulation Assays

The assessment of the patient’s coagulation status included first- and second-level haemostasis tests. Apart from a mild prolongation of the aPTT, the classic coagulation assays showed no significant alterations (Table 2).

**Figure 1:**
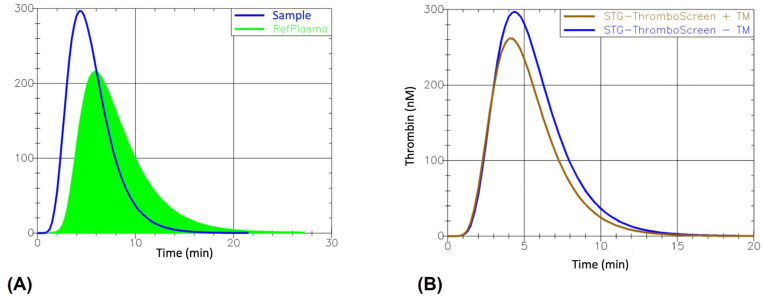
Thrombin generation curves obtained from athletes’ plasma using the ThromboScreen assay. (**A**) TGA curve measured in the absence of TM. TGA parameters are related to a reference plasma and expressed as ratios (temporal data) or percentages (thrombin concentration-related data). (**B**) TGA curve measured in the presence of TM. TM, thrombomodulin.

Given the athlete’s thrombotic manifestation, for a more global view of the patient’s haemostatic asset, we decided to perform a Thrombin Generation Assay (TGA), whose curves highlighted an alteration of the parameters obtained in the absence and presence of thrombomodulin (Figure 1).

At the same time, the slight lengthening of the aPTT and the minor decrease in FVIII activity induced us to further investigate the von Willebrand panel, which showed a reduction in vWF Antigen (vWF:Ag), vWF Ristocetin Cofactor Activity (vWF:RCo), vWF Collagen Binding (vWF:CB). All the results are shown in Table 2. 

Normalized values for lag time, peak height, time to peak, endogenous thrombin potential (ETP), and Velocity Index were collected using the ThromboScreen assay in the absence of thrombomodulin (TM). The ETP inhibition parameter has been calculated from ETP values obtained in the presence and absence of TM (Table 3).

### 2.4. Genetic Tests

**Figure 2:**
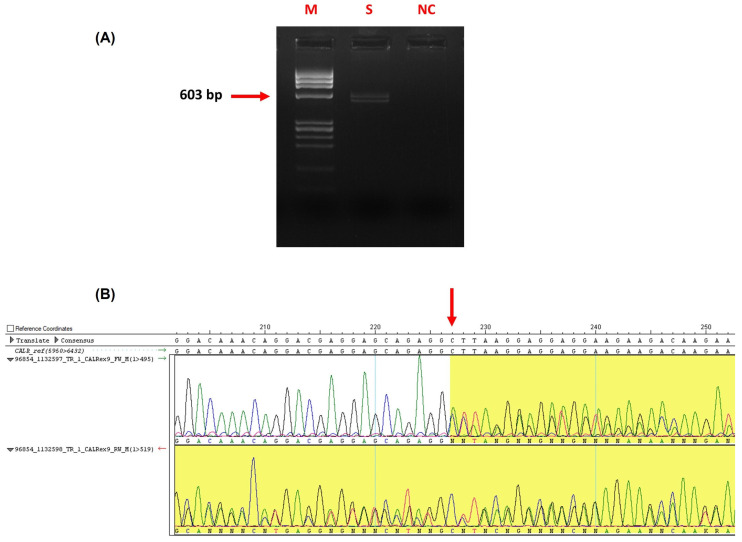
*CALR* mutation identified in the analysed athlete. (**A**) Agarose gel electrophoresis showing the difference in length of the two alleles, one being wild type and the other carrying the 52-bp deletion. (M: Marker IX, S: Sample, NC: Negative Control). (**B**) Sanger electropherogram represents the frameshift caused by the 52-bp deletion.

Based on the results of the biochemical, haematological and coagulation parameters, a diagnosis of acquired von Willebrand disease secondary to Essential Thrombocythemia (ET) was hypothesized. To confirm this hypothesis and unveil the mechanisms underlying the observed thrombotic event, specific molecular tests were carried out. In particular, molecular analyses were performed to evaluate the most common mutations known to be associated with ET. First, *JAK2* exon 14 sequence analysis was carried out. Indeed, *JAK2* exon 14 is mutated in about 60% of all ET cases, the mutation c.1849 G > T p.(Val617Phe) being the most common. However, no variants were found in our case. Thus, *CALR* exon 9 and *MPL* exon 10 were also sequenced, taking into account that their mutations explain 30% and 3–8% of all cases of the disease, respectively. Even if no variants were found in *MPL* gene exon 10, one variant was instead identified in the coding region of *CALR* exon 9. In detail, a 52-bp deletion that is known to be responsible for more than 80% of all *CALR* mutations was identified. Interestingly, the agarose gel electrophoresis analysis allows the identification of the deletion in heterozygous status since the pattern of migration highlights two different bands, differing from the deletion-related base pair length (Figure 2A). The mutation’s presence was then confirmed by Sanger sequencing electropherogram analysis (Figure 2B). This deletion, namely c.1099_1150del p.(Leu367ThrfsTer46) causes frameshift with a premature protein stop codon and is classified as “pathogenic” according to the ACMG guidelines.

Moreover, to further support this finding and exclude a thrombophilic status, we also searched for the following mutations: Leiden-type mutation of the *Factor V* gene (FVL), G20210A mutation of the prothrombin gene, and C677T mutation of the *5,10-methylenetetrahydrofolate reductase* (*MTHFR*) gene. All the above-mentioned mutations resulted in wild-type results in the tested athlete (data not shown). Thus, all together, the results obtained allowed to clarify the molecular mechanisms responsible for the observed thrombotic event and confirmed the hypothesised diagnosis of ET in this athlete.

## 3. Discussion

In recent years, laboratory medicine has assumed considerable importance in sports medicine, providing a valid tool for monitoring the athlete’s health. In fact, it is known that practicing sports at agonist levels exposes athletes to an increased risk of infections, inflammation, muscle damage, and cardiovascular disorders, which can be seriously harmful if not promptly treated.

A 28-year-old male athlete was admitted to our Haematology department following a thrombotic event. A biochemical, haematological, and coagulation check-up showed a normal leukocyte count, a slightly reduced erythrocyte count, and normal levels of haemoglobin and haematocrit, but a marked thrombocytosis. To rule out that the increase in the number of platelets was due to a reactive nature or iron deficiency anaemia, as reported by Bleeker et al. [24], we measured the concentrations of CRP, ferritin, and iron, all of which were within the normal range. First-level haemostasis analysis showed a normal range for fibrinogen, D-dimer and PT but a slight prolongation of the aPTT. Since no other reasons that could explain the lengthening of the aPTT emerged and because the athlete was not taking any anticoagulant drugs, considering the widely known correlation between the increase in the number of platelets and AvWD [18], a screening test for von Willebrand disease was performed. Surprisingly, the von Willebrand panel displayed a reduction of vWF:Ag, vWF:RCo, vWF:CB and a mild decrease in FVIII, with a pathological vWF:Rco/vWF:Ag ratio of 0.63 and a borderline normal vWF:CB/vWF:Ag ratio of 0.74. Therefore, based on biochemical data, we directed our laboratory diagnosis towards a possible AvWD secondary to ET, confirmed by a negative personal and familial anamnesis for haemorrhagic events. The reduction of vWF in the acquired form of VWD is complex and underlies several pathophysiological mechanisms: antibodies directed against the vWF, absorption of vWF by platelets or malignant cells, high shear stress conditions, or increased proteolysis of vWF [14]. To exclude any antibody evolution direct against the vWF and pinpoint the cause of the reduction of vW:Ag mixing studies were performed on a 1:1 mixture of the patient’s plasma and normal pool plasma, revealing the complete correction of the vW:Ag test. This comes from the fact that mechanisms likely involved in the pathogenesis of AvWD in ET are linked to the increase in proteolysis by the ADAMTS-13 enzyme, inducing a loss of large VWF multimers; the intensity of this loss is inversely proportional to the number of platelets [25]. Nevertheless, since our athlete never had a hemorrhagic phenotype but presented a thrombotic event, thrombophilia screening was carried out. The screening didn’t reveal any significant alteration, despite the presence of natural coagulation inhibitors, APS screening, and APC resistance. Molecular analyses excluded the presence of a thrombophilic status since the FVL, the *prothrombin* G20210A, and the *MTHFR* C677T mutations were not present in the tested athlete. To further investigate the patient’s pro-thrombotic state, we measured the thrombin generation of the athlete by TGA, which is a global hemostasis assay investigating, in platelet-poor plasma, both thrombin formation and inhibition. Classical coagulation tests, such as PT and PTT, are sensitive to procoagulant factor deficiencies but not to reductions in the anticoagulant counterpart, making it difficult to detect hypercoagulable states [26]. On the other hand, TGA allows for a global view of the in vivo haemostatic situation, considering both procoagulant and anticoagulant forces. Data obtained by TGA highlighted a hypercoagulable state due to an increase in peak height, ETP, velocity index and a reduction of lag time and time to peak. Hypercoagulability was most evident when TM was added to the patient’s plasma. The latter, acting as a co-factor of thrombin in the activation of the protein C-pathway, inhibit the procoagulant function of thrombin and should decrease the ETP of the thrombogram in a normal individual. In our case, the ineffectiveness of the TM-PC-PS system was highlighted, as shown by the decrease in ETP inhibition. The enhancement of thrombin production highlighted through TGA on the athlete plasma is certainly not directly linked to the increase in platelets, since this test is performed on a platelet-poor plasma, but can be explained by the activation of platelets that is observed in ET patients. This is caused by the exposure of phospholipids present on the surface of platelets to tenase and prothrombinase complexes; moreover, activated platelets can express anionic phospholipids and TF on their surface, thereby providing the conditions for stimulating the coagulation cascade [27]. Furthermore, it is well known that athletes are frequently exposed to several conditions associated with an increased risk of thrombosis, including long-haul travel, dehydration, trauma, post-exercise haemoconcentration, and high-intensity exercise, which exposes them to an increase in hypercoagulability compared to a normal individual [28]. All these aspects may have contributed to the coagulation derangement in this athlete, inducing an increase in thrombin generated and leading to thrombotic event. The results obtained by this study confirm how ET is characterised by a wide range of clinical manifestations, ranging from thrombotic to haemorrhagic manifestations. Thrombotic risk should also be taken into account in these patients, particularly in subjects such as athletes, who already have an imbalance of the haemostatic system generated by the transient increase in blood clotting, platelet clumping and fibrinolytic activity due to intense physical exercise [28]. Thus, performing TGA in athletes with a family or personal history of thrombotic events may be useful, particularly in the case of athletes with a positive history of a thrombotic event but without alterations highlighted by classic coagulation tests (as in our case). Anyway, the extensive use of TGA in all athletes, regardless of their clinical background, should be avoided, taking into account both the appropriateness of the laboratory tests and the expense related to the test.

Mutated genes responsible for ET are Janus kinase 2, calreticulin and thrombopoietin receptor, with a frequency of 60%, 30%, and 3%, respectively. Moreover, 10–20% of ET patients, called Triple Negative, do not carry any of the three driver mutations. Based on the previous findings and using molecular approaches, we found a 52-bp deletion in exon 9 of the *CALR* gene, known as the *CALR* type 1 mutation, responsible for the generation of a frameshift mutation determining the alteration of the amino acid sequence at the C-terminal of the protein. The mutation causes the replacement of negatively charged with positively charged or neutral amino acids [29]. Calreticulin resides in the lumen of the endoplasmic reticulum, and its C-terminal domain is responsible for calcium buffering activity, which controls calcium homeostasis [30]. According to Kim et al., mutant *CALR* has been suggested to induce myeloproliferative neoplasm via interactions with the thrombopoietin receptor (MPL): in fact, the oncogenicity of mutant *CALR* is thought to depend on the positive charge of the C-terminus, which is necessary for the physical interaction between mutant *CALR* and *MPL* [30]. Indeed, mutant *CALR* is accountable for a gain-of-function property, triggering thrombopoietin receptor activation and downstream cellular signalling via the JAK-signal transducer and activator of transcription (JAK-STAT) pathway. Despite all of this evidence, making *CALR* one of the most important diagnostic markers tested upon diagnosis of a myeloproliferative disorder, our data suggest a milder disease in patients harbouring *CALR* mutations since it is related to higher platelet counts, lower haemoglobin levels, and leukocyte counts but a lower risk of thrombosis than patients with *JAK2* and *MPL* mutations [31].

## 4. Materials and Methods

### 4.1. Ethical Approval

The study was conducted according to the ethical guidelines of the Helsinki Declaration of the World Medical Association and was approved by the ethics committee (protocol 200/17) of the University of Naples Federico II. The athlete provided written consent to carry out biochemical laboratory tests and genetic analysis.

### 4.2. Clinical Laboratory Determinations

Blood samples were taken in the morning (8:00 a.m.) before training, after 72 h of rest from training. As a safeguard measure, blood, serum, and plasma samples were frozen at −80 °C in case any analysis had to be repeated. Standard biochemical analyses were performed using a standard serum analyser in Architect c16000 (Abbott Diagnostics, Chicago, IL, USA). Red blood cell and platelet counts were performed through the Siemens Advia 2120i haematology analyser. All analyses were performed in duplicate in order to guarantee the accuracy of the results.

All the procedures took place according to the manufacturer’s recommendation.

### 4.3. Coagulation Assay

Standard coagulation analyses evaluating first and second-level determinations were assessed using an ACLTOP 550CTS coagulometer system (Instrumentation Laboratory Company, Bedford, MA, USA). Results obtained for aCL and aβ2GPI IgG and IgM and the von Willebrand parameters were carried out with a fully automated quantitative chemiluminescent immunoassay by HemosIL^®^ AcuStar (Instrumentation Laboratory Company, Bedford, MA, USA). To better understand the global coagulation process, we measured thrombin generation by TGA on a platelet-poor plasma sample with the aid of the ST Genesia^®^ Automated TG Tool (STG, Stago, Asnières-sur-Seine, France), using the STG^®^-ThromboScreen (STG-TS) reagent in the presence and absence of thrombomodulin (TM). TGA is a comprehensive assay for haemostasis evaluation based on the analysis in platelet-poor plasma of both thrombin formation and inhibition that allows, as a result, to evaluate the effects of plasma pro- and anticoagulant factors. The following TG parameters were recorded and investigated: lag time, peak height, time to peak, start tail, ETP, and ETP inhibition mediated by TM. All the procedures took place according to the manufacturer’s recommendation.

### 4.4. Molecular Analyses

One genomic DNA sample was obtained from a peripheral blood sample in EDTA by standard procedures (Promega, Madison, WI, USA) and processed for a quantitative check using the NanoDrop spectrophotometer instrument (Thermo Fisher Scientific Inc., Waltham, MA, USA).

*JAK2*, *CALR,* and *MPL* gene genomic sequences were downloaded from the Ensemble genome browser (https://www.ensembl.org/index.html, accessed on 1 January 2023), choosing all of them with the canonical transcript. Custom primer pairs were designed to specifically amplify *JAK2* exon 14, *CALR* exon 9, and *MPL* exon 10, respectively, by using the web-based tool Primer3 Input (https://primer3.ut.ee/, accessed on 1 January 2023) and checked for amplification specificity through the web-based tool Primer Blast (https://www.ncbi.nlm.nih.gov/tools/primer-blast, accessed on 1 January 2023) (Table 4).

Polymerase Chain Reaction (PCR) experiments were performed using the AccuStart™ PCR Super Mix (VWR International, Radnor, PA, USA) following the manufacturer’s instructions. Amplicons qualitative analysis was carried out by electrophoresis on a 3% agarose gel. 

Direct sequencing was performed for each PCR product with the ABI 3100 capillary sequencer (Applied Biosystems Inc., Foster City, CA, USA), and sequence data analysis was carried out using the SeqMan Pro programme (DNASTAR, Inc., Madison, WI, USA). Next, DNA variants were categorised according to Ensembl (https://www.ensembl.org/index.html, accessed on 1 February 2023), ClinVar (https://www.ncbi.nlm.nih.gov/clinvar/, accessed on 1 February 2023) and dbSNP (https://www.ncbi.nlm.nih.gov/SNP/, accessed on 1 February 2023) databases, and the possible impact of specific variations at the protein level were predicted using the VarSome (https://varsome.com, accessed on 1 February 2023) tool.

## 5. Conclusions

An appropriate functional and clinical evaluation, possibly including some major laboratory investigations, should be recommended to anyone wishing to begin an exercise program. Furthermore, continuous and correct monitoring of the health of athletes, especially at elite levels, is suggested in order to avoid muscle damage or even the appearance of pathological conditions that could endanger the athlete’s life. In this context, the case reported herein underlines once again the need for integrated, multidisciplinary approaches, including biochemical, haematological, immunological, and molecular analyses, in order to clarify the bases of clinical signs and diseases and provide useful information to support athletes’ management and avoid the onset of diseases that could also be fatal, as recently reported. In particular, based on clinical observation, biochemical, haematological and coagulation evaluations, and molecular tests, we were able to highlight the molecular mechanisms related to the onset of the observed phenotype. This, in turn, will not only improve the management of the analysed athlete but may also provide a model for a more appropriate and multidisciplinary evaluation of elite athletes, including molecular aspects.

## Figures and Tables

**Table 1:** Biochemical and haematological parameters of the athlete.

Parameters	Results
Iron (65–175 µg/dL) *	143
Ferritin (22–275 ng/dL) *	90
C-reactive protein (CRP) (0–5 mg/dL) *	0.3
Leucocytes (4.8–10.8 × 103/µL) *	6.43
Erytrocytes (4.2–5.6 × 106/µL) *	3.94 ↓
Haemoglobin (12–17.5 g/dL) *	14.1
Haematocrit (37–54%) *	39.8
Platelets (130–400 × 103/µL) *	898 ↑

* Reference values of the Italian Society of Clinical Biochemistry and Clinical Molecular Biology (SIBioC); ↓,measure lower respect to the reference values; ↑, measure higher respect to the reference values.

**Table 2:** Coagulation parameters.

Parameters	Results
Prothrombin Time (PT) (0.8–1.20 Ratio) **	1.18
Activated Partial Thromboplastin Time (aPTT) (0.8–1.20 Ratio) **	1.21 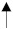
FIBRINOGEN (160–350 mg/dL) **	207
D-DIMER (0–500 ng/mL) **	424
ANTITHROMBIN III (70–120%) **	107
Lupus anticoagulant (LAC) (0.8–1.20 Ratio) **	0.98
Anti-Cardiolipin IgG (<20 U/mL) **	7.8
Anti-Cardiolipin IgM (<20 U/mL) **	1.1
Anti-β2 glycoprotein I IgG (<20 U/mL) **	3.9
Anti-β2 glycoprotein I IgM (<20 U/mL) **	0.3
APC Resistance (>0.75 NTR) **	1.04
Protein C (>62%) **	88
Free Protein s (>58%) **	103
Factor VIII (FVIII) (50–130%) **	57
Factor IX (FIX) (50–120%) **	76
Factor XI (FXI) (50–120%) **	95
Factor XII (FXII) (50–120%) **	67
vWF:Ag (Blood type 0 44–116% non–0 Blood type 63–159%) **	33.5 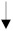
vWF:RCo (Blood type 0 44–116% non–0 Blood type 63–159%) **	21.3 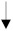
vWF:CB (Blood type 0 44–116% non–0 Blood type 63–159%) **	24.8 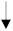

** Reference values were defined based on a healthy population; 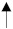, measure higher respect to the reference values; 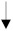, measure lower respect to the reference values.

**Table 3:** TGA data measured by ST Genesia using the ThromboScreen assay. Hypercoagulable state is evidenced by thrombogram parameters due to the reduction of lag time, time to peak and ETP inhibition and increase in peak height, ETP, and velocity Index.

TGA Parameters	Results
Lag Time (1.1–1.3 ratio) °	0.88 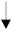
Peak Height (45–66%) °	125.7 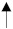
Time To Peak (1.2–1.3 ratio) °	0.97 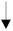
ETP (59–80%) °	100.4 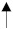
ETP Inhibition (60–73%) °	16.05 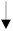
Velocity Index (36–57%) °	124 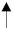

° Reference values obtained from Calzavarini et al. [23] 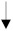, measure lower respect to the reference values; 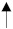, measure higher respect to the reference values.

**Table 4:** Full list of the primers sequences used to amplify *JAK2*, *CALR,* and *MPL* genes.

Gene	Transcript	Exon	Forward 5′->3′	Reverse 5′->3′	Product Lenght (bp)
*JAK2*	NM_004972.4	14	GGTTTCCTCAGAACGTTGATGG	TTGTTTGGGCATTGTAACCTTCT	492
*CALR*	NM_004343.4	9	CAAGTCTGGCACCATCTTTGAC	AGGAGGGGAACAAAACCAAAATC	524
*MPL*	NM_005373.3	10	TAGGGGCTGGCTGGATGAG	ACAGAGCGAACCAAGAATGC	250

## Data Availability

Data are contained within the article.
